# Renoprotective effects of extracellular fibroblast specific protein 1 via nuclear factor erythroid 2-related factor-mediated antioxidant activity

**DOI:** 10.1038/s41598-023-49863-y

**Published:** 2023-12-18

**Authors:** Naoki Takahashi, Seiji Yokoi, Hideki Kimura, Hironobu Naiki, Taiji Matsusaka, Yasuhiko Yamamoto, Kimihiko Nakatani, Kenji Kasuno, Masayuki Iwano

**Affiliations:** 1 Department of Nephrology, Faculty of Medical Sciences, University of Fukui, 23-3 Matsuokashimoaizuki, Eiheiji-Cho, Yoshida-Gun, Fukui, 910-1193 Japan; 2 Department of Clinical Laboratory, Faculty of Medical Sciences, University of Fukui, Fukui, Japan; 3 Department of Molecular Pathology, Faculty of Medical Sciences, University of Fukui, Fukui, Japan; 4 Institute of Medical Sciences and Department of Basic Medicine, Tokai University School of Medicine, Kanagawa, Japan; 5 Department of Biochemistry and Molecular Vascular Biology, Kanazawa University Graduate School of Medical Sciences, Kanazawa, Japan; 6 Department of Nephrology, Yamashiro General Medical Center, Kizugawa, Kyoto Japan

**Keywords:** Medical research, Nephrology

## Abstract

Podocyte expression of fibroblast specific protein 1 (FSP1) is observed in various types of human glomerulonephritis. Considering that FSP1 is secreted extracellularly and has been shown to have multiple biological effects on distant cells, we postulated that secreted FSP1 from podocytes might impact renal tubules. Our RNA microarray analysis in a tubular epithelial cell line (mProx) revealed that FSP1 induced the expression of heme oxygenase 1, sequestosome 1, solute carrier family 7, member 11, and cystathionine gamma-lyase, all of which are associated with nuclear factor erythroid 2-related factor (Nrf2) activation. Therefore, FSP1 is likely to exert cytoprotective effects through Nrf2-induced antioxidant activity. Moreover, in mProx, FSP1 facilitated Nrf2 translocation to the nucleus, increased levels of reduced glutathione, inhibited the production of reactive oxygen species (ROS), and reduced cisplatin-induced cell death. FSP1 also ameliorated acute tubular injury in mice with cisplatin nephrotoxicity, which is a representative model of ROS-mediated tissue injury. Similarly, in transgenic mice that express FSP1 specifically in podocytes, tubular injury associated with cisplatin nephrotoxicity was also mitigated. Extracellular FSP1 secreted from podocytes acts on downstream tubular cells, exerting renoprotective effects through Nrf2-mediated antioxidant activity. Consequently, podocytes and tubular epithelial cells have a remote communication network to limit injury.

## Introduction

A member of the S100 family of calcium-binding proteins, fibroblast-specific protein 1 (FSP1), also known as S100A4 and Mts1, is constitutively produced in fibroblasts and metastatic cancer cells^1,2^. FSP1 has been largely investigated in relation to cancer due to its identification as a metastasis promoter. These investigations demonstrated that FSP1 enhances tumor cell invasion and serves as a key prognostic indicator in a variety of cancer types^3^. However, FSP1 also participates in a variety of regular biological processes, such as fibrosis, inflammation, angiogenesis, and neuroprotection^4^, and ablating fibroblasts expressing FSP1 (FSP1 + fibroblasts) during fibrogenesis markedly reduces the fibrotic area in a mouse obstructed kidney model^5^. Therefore, the development of interstitial fibrosis may be aided by FSP1 + fibroblasts in the renal interstitium. Furthermore, the number of FSP1 + fibroblasts can serve as a predictor of IgA nephropathy^6^.

Within the glomeruli of humans, podocytes express FSP1 in cases of IgA nephropathy, diabetic nephropathy, lupus nephritis and ANCA-associated glomerulonephritis^7,8^, and most detached podocytes in urine collected from patients with diabetic nephropathy express FSP1^8^. The numbers of FSP1 + podocytes are elevated in mice treated with adriamycin (ADR), a model of human focal and segmental glomerulosclerosis (FSGS), and they positively correlate with the severity of proteinuria and glomerulosclerosis. Furthermore, ADR-induced FSGS and associated proteinuria are markedly reduced in FSP1 deletion mice^9^. These results imply that podocyte detachment is related to podocyte epithelial mesenchymal transition. However, in patients with active glomerulonephritis, most FSP1 + podocytes do not detach from the glomerular basement membrane, suggesting that FSP1 expression alone is not enough to induce podocyte detachment, and the functions of FSP1 in podocytes remain unclear.

FSP1 is also secreted from FSP1-expressing cells, and extracellular FSP1 promotes cell survival, differentiation, proliferation, motility, invasion, and angiogenesis, among other biological processes. While many actions of FSP1 have been reported to exacerbate tissue damage through induction of fibrosis and inflammation, actions of FSP1 that provide neural and myocardial protection have also been reported^1,2,4^. However, research has not yet looked at the effects of extracellular FSP1 on renal tissue. We hypothesized that extracellular FSP1 secreted from FSP1 + podocytes should have some effects on renal tissue. Using a model of cisplatin-induced acute kidney injury, we investigated the effects of extracellular FSP1 on tubular epithelial cells (TEC). Our findings suggest that extracellular FSP1 plays a novel cytoprotective role and that podocytes can interact with TECs through extracellular FSP1.

## Results

### FSP1 exerts cytoprotective effects via Nrf2-induced antioxidant activity

**Figure 1:**
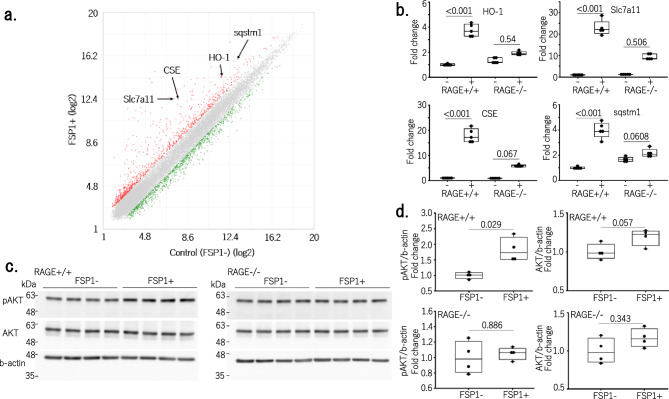
FSP1 enhances the expression of factors associated with Nrf2 activation in TECs in a RAGE-dependent manner. (**a**) A microarray result. Red dots are genes whose expression levels are more than double by the administration of FSP1 (FSP1+), and green dots are genes whose expression levels were less than half by the administration of FSP1. (**b**) Quantitative real-time PCR analysis of mRNA showed that heme oxygenase 1 (HO-1), solute carrier family 7, member 11 (Slc7a11), cystathionine gamma-lyase (CSE), and sequestosome 1 (sqstm 1) were all up-regulated in mProx cells treated with FSP1. These effects were blocked after RAGE knockout (RAGE^−/−^) in mProx cells (n = 5 in each group). (**c**) Western blots showing AKT and pAKT levels in wild-type and RAGE^−/−^ mProx cells treated with or without FSP1. (**d**) Densitometric analysis of the blots. FSP1 significantly increased phosphorylated AKT levels (P = 0.029) in a RAGE-dependent manner (n = 4 in each group).

We first investigated the effects of recombinant FSP1 protein on gene expression profile of tubular epithelial cell line (mProx) cells. Among the 142 genes whose expression was upregulated more than 5-fold by microarray analysis, we found four factors associated with Nrf2 activation: heme oxygenase 1 (HO-1), sequestosome 1 (p62, sqstm 1), solute carrier family 7, member 11 (Slc7a11), and cystathionine gamma-lyase (CSE) were upregulated 6.54-, 6.73-, 33.82-, and 24.59-fold, respectively (Fig. 1a, Supplementary Table). qPCR revealed that these four factors were significantly induced in mProx cells by FSP1 (P < 0.001) (Fig. 1b). Both sqstm 1 and CSE contribute to Nrf2 activation by inducing Nrf2 dissociation from Keap1 and enhancing Nrf2 nuclear translocation^10,11^. Nrf2 activation stimulated the expression of Nrf2-targeted downstream genes, including HO-1 and Slc7a11^12,13^. Simultaneous up-regulation of these four factors strongly suggests that FSP1 enhances Nrf2 activation. In addition, FSP1 enhanced AKT phosphorylation, which is involved in the essential signaling pathway that leads to Nrf2 activation^14^ (Fig. 1c,d, Supplementary Fig. 1a,b).

**Figure 2:**
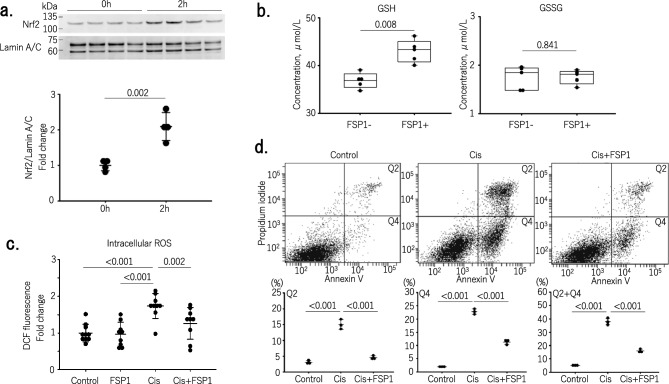
Cytoprotective effects of FSP1 through Nrf2-mediated antioxidant activity in TECs. (**a**) Upper: Western blots of mProx cell nuclei before incubation with FSP1 (0 h) and after 2 h incubation with FSP1 (2 h). Nuclear translocation induced by FSP1 of Nrf2 was clearly observed. Lower: Densitometric analysis of the blots (n = 4 in each group). (**b**) FSP1 increased reduced glutathione (GSH) levels in mProx cells, while oxidized glutathione (GSSG) levels were unchanged (n = 5 in each group). (**c**) Fold change in dichlorodihydrofluorescein (DCF) fluorescence by the addition of FSP1 only (FSP1), cisplatin only (Cis), or FSP1 and cisplatin (Cis + FSP1). FSP1 significantly inhibited cisplatin-induced increases in DCF fluorescence by reactive oxygen species (ROS) production in mProx cells (n = 9 in each group) (P = 0.002). (**d**) Incidence of apoptosis among mProx cells without any treatments (control), treated with cisplatin (Cis) with or without FSP1. The numbers of apoptotic cells were significantly lower among the FSP1 treated cells (n = 3 in each group) (P < 0.001).

The receptors of advanced glycation end products (RAGE) has been identified as the receptor for extracellular FSP1^15^. Therefore, we established a RAGE^−/−^ mProx cell line using the CRIPR/Cas9 system. Upregulation of HO-1, sqstm 1, Slc7a11, and CSE by FSP1 as well as phosphorylation of AKT were all reduced in RAGE^−/−^ cells, confirming that these effects on TECs were induced, at least in part, by FSP1 (Fig. 1b–d). Furthermore, western blot analysis further confirmed that FSP1 induced Nrf2 translocation, thus increasing its localization within the nucleus of mProx cells (Fig. 2a, Supplementary Fig. 2).

Glutathione reductase is known to be a Nrf2-targeted downstream gene^11^. We confirm that after FSP1 administration reduced glutathione (GSH) levels increased significantly (P = 0.008) while oxidized glutathione (GSSG) levels were unchanged (Fig. 2b). Because Nrf2 activation functions as a critical regulator of a cell’s defense against oxidative stress, we next used dichlorofluorescein assays to determine whether FSP1 suppresses intracellular ROS levels. We found that FSP1 significantly reduced ROS levels in cisplatin-treated mProx cells (P = 0.002) (Fig. 2c). Annexin V/PI staining and FACS analysis were then carried out to determine whether FSP1 prevents cisplatin-induced cell death. Cisplatin treatment resulted in the induction of both early (Annexin V positive/PI negative) and late (Annexin V positive/PI positive) cell death. FSP1 significantly reduced the levels of both types of cell death (P < 0.001) (Fig. 2d). In addition, western blot analysis confirmed that FSP1 diminished cleaved caspase-3 expression induced by cisplatin exposure in mProx cells (Supplementary Fig. 3).

### Renoprotective effects of FSP1 in cisplatin nephrotoxicity

**Figure 3:**
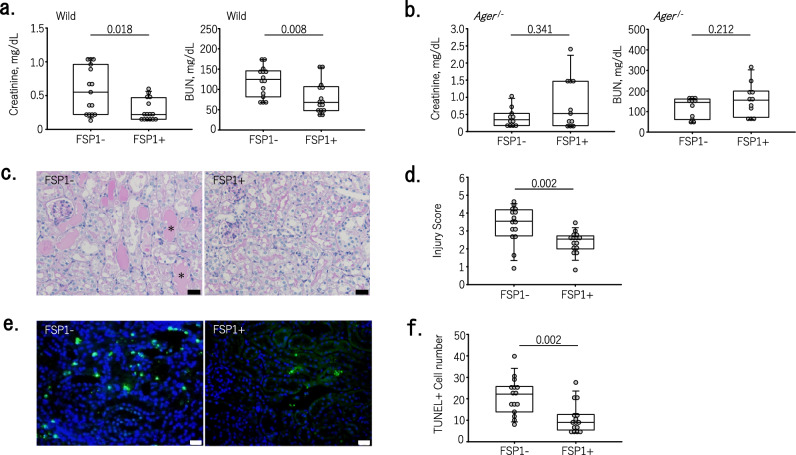
Intraperitoneal administration of FSP1 ameliorates acute kidney injury observed in cisplatin nephrotoxicity. (**a**) Intraperitoneal administration of FSP1 significantly suppressed cisplatin-induced increases in serum creatinine (P = 0.018) and BUN (P = 0.008) levels (n = 15 in each group). (**b**) In *Ager*^−/−^ mice, intraperitoneal administration of FSP1 did not suppress cisplatin-induced increases in serum creatinine and BUN (n = 11 in each group). (**c**) Representative micrographs of the renal cortices of mice treated with cisplatin with or without FSP1. Sections were stained with periodic acid-Schiff (PAS). Damaged tubules with diffuse tubular dilatation and intraluminal casts (asterisks) were observed in mice with cisplatin without FSP1. Bars = 20 μm. (**d**) Acute tubular injury scores calculated as described in methods section. Intraperitoneal administration of FSP1 significantly improved cisplatin-induced tubular damage (number of animals = 15 in each group) (P = 0.002). (**e**) Representative photomicrographs showing TUNEL-positive cells in renal cortices from mice treated with cisplatin with or without FSP1. Bars = 20 μm. (**f**) Numbers of TUNEL-positive cells. Intraperitoneal administration of FSP1 significantly decreased the number of TUNEL positive cells (number of animals = 15 in each group) (P = 0.002).

To confirm the cytoprotective effects of FSP1 in vivo, we tested whether FSP1 would attenuate cisplatin-induced nephrotoxicity. Intraperitoneal administration of FSP1 significantly improved Scr (P = 0.018) and BUN (P = 0.008) levels in wild-type mice with cisplatin nephrotoxicity (Fig. 3a). Those improvements in kidney function after FSP1 administration were lost in RAGE knockout mice (*Ager*^−/−^ mice) (Fig. 3b). FSP1 also reduced tubular injury scores, as well as cell death indicated by terminal deoxynucleotidyl transferase dUTP nick-end labeling (TUNEL), in mice with cisplatin nephrotoxicity (Fig. 3c–f). These findings suggest that FSP1 may exert protective effects against cisplatin-induced nephrotoxicity.

**Figure 4:**
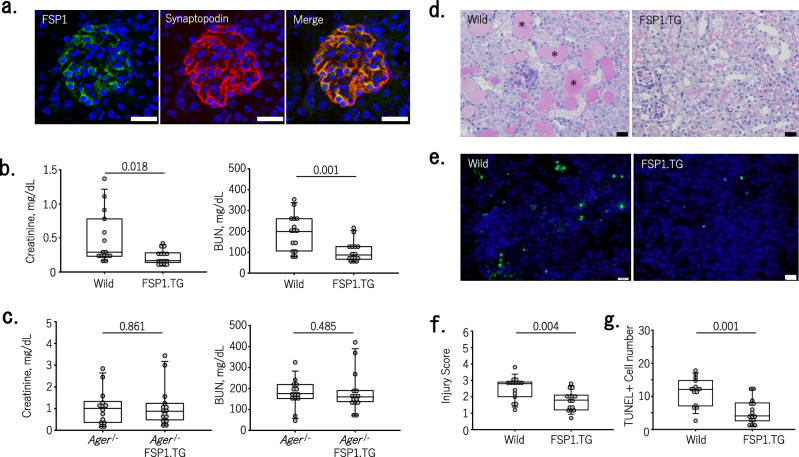
The expression of FSP1 podocytes ameliorated acute kidney injury observed with cisplatin nephrotoxicity. (**a**) In FSP1 transgenic (FSP1.TG) mice in which FSP1 (green) was specifically expressed in podocytes, FSP1 was colocalized with the podocyte marker synaptopodin (red). Nuclei were stained with DAPI (4ʹ,6-diamidino-2ʹ-phenylindole; blue). Bars = 20 μm. (**b**, **c**) Cisplatin-induced increases in serum creatinine and BUN were suppressed in FSP1.TG mice but not in FSP1.TG backcrossed with *Ager*^−/−^ mice (number of animals = 15 in each group). (**d**, **f**) Representative micrographs showing periodic acid-Schiff (PAS) stained mouse renal cortex (**d**) and tubular injury scores (**f**) showing that tubular damage is improved in FSP1.TG mice treated with cisplatin (number of animals = 15 in each group). Damaged tubules with diffuse tubular dilatation and intraluminal casts (asterisks) were observed in wild-type mice with cisplatin. Bars = 20 μm. (**e**, **g**) Representative micrographs showing TUNEL positivity (green) in the renal cortex of cisplatin-treated mice (**e**) and the number of TUNEL-positive cells (**g**). The number of TUNEL-positive cells decreased significantly in FSP1.TG mice (number of animals = 15 in each group) (P = 0.001). Nuclei were stained with DAPI (4ʹ,6-diamidino-2ʹ-phenylindole; blue). Bars = 20 μm.

Finally, to mimic human glomerulonephritis in which podocyte expression of FSP1 is observed, we generated transgenic (FSP1.TG) mice in which podocyte FSP1 expression was driven by the nephrin promoter (Fig. 4a). We established two lines of FSP1.TG mice and chose the high expressor for this study. We first examined the baseline effects of FSP1 overexpression on kidney biology and NRF2 activity. As shown in Supplementary Fig. 4a, there were no differences in renal function (serum creatinine and BUN) or albuminuria between wild-type and FSP1.TG mice. Levels of Slc7a11 and HO-1 mRNA expression were significantly elevated in renal cortex of FSP1.TG mice; however, mRNA expression of cystathionine and seqestosome 1 were not (Supplementary Fig. 5). FSP1 was expressed in all podocytes from FSP1.TG mice and in resident fibroblasts from both wild-type and FSP1.TG mice (Supplementary Fig. 4b). There were no differences in FSP1-positive areas (except glomeruli) among wild-type mice, *Ager*^−/−^ mice and FSP1.TG mice.

Kidney function was improved in FSP1.TG mice with cisplatin nephrotoxicity compared to wild-type mice (Fig. 4b). By abolishing RAGE expression through backcrossing FSP1.TG with *Ager*^−/−^ mice, the improvement observed in FSP1.TG mice was lost (Fig. 4c). Furthermore, tubular injury scores, cleaved caspase-3 expression, and cell death indicated by TUNEL were all reduced in FSP1.TG mice (Fig. 4d–g, Supplementary Fig. 6). These beneficial effects of overexpressing FSP1 in podocytes resembled those observed after FSP1 administration. After cisplatin exposure, FSP1-positive cells and areas did not differ, except in FSP1 + podocytes. This suggests the source of extracellular FSP1 is FSP1 + podocytes (Supplementary Fig. 7).

## Discussion

We had hypothesized that extracellular FSP1 secreted from FSP1 + podocytes should have some effects on renal tissue. In this study, we determined that FSP1 exerts cytoprotective effects on TEC through Nrf2-induced antioxidant activity in vitro and reduces levels of cleaved caspase-3 expression in vitro and in vivo. This suggests FSP1 has an anti-apoptotic effect on TEC. FSP1 ameliorated acute tubular injury in mice with cisplatin nephrotoxicity, which is a representative model of ROS-mediated tissue injury^16,17^. In transgenic mice that express FSP1 specifically in podocytes, tubular injury associated with cisplatin nephrotoxicity was also improved. These results highly support our hypothesis that extracellular FSP1 secreted from FSP1 + podocytes exerts cytoprotective effects on TEC in vivo and suggest the existence of interaction between podocytes and TEC via extracellular FSP1 in vivo.

The Nrf2-Keap1 pathway acts as a crucial regulator of a cell's defense against oxidative stress by controlling the expression of various protective cellular proteins^18^. Here, we newly identified four factors associated with Nrf2 activation that were induced by FSP1 in TEC. First, we observed a more than tenfold increase in CSE expression. A major product of the CSE-catalyzed reaction is hydrogen sulfide (H_2_S), which exerts anti-inflammatory, anti-oxidative stress, and anti-fibrotic effects^18^. Furthermore, H_2_S donors have been reported to reduce cisplatin-induced kidney injury^19^. Although we did not examine H_2_S generation in the present study, it is likely that H_2_S production is associated with the renoprotection observed in this study. H_2_S induces S-sulfhydration of Keap1 and activation of Nrf2 in association with oxidative stress^20^, and overexpression of CSE reportedly reduces ROS levels^21^. Second, we observed a > 20-fold increase in Slc7a11, a downstream target gene of Nrf2 responsible for inhibiting ferroptosis. Ferroptosis plays a key role in cisplatin-induced acute kidney injury^22^. This suggests that the renoprotective effects of FSP1 may be achieved, in part, by inhibiting ferroptosis via Slc7a11 overproduction, although ferroptosis was not examined in this study. Third, there was a > 3-fold increase of sqstm1, which interacts with the Nrf2 binding site on Keap1, a component of Cullin-3-type ubiquitin ligase for Nrf2. Therefore, overproduced sqstm1 would competitively inhibit the interaction between Nrf2 and Keap1, resulting in the stabilization of Nrf2 and the transcriptional activation of Nrf2 target genes^10^. Induction of the sqstm 1 gene by oxidative stress is mediated by Nrf2 and, at the same time, sqstm 1 contributes to the activation of Nrf2^23^. Fourth, there was a > 3-fold increase in HO-1, which is a representative target of Nrf2 activation. The activation of the Nrf2/HO-1 pathway is essential for protection against cisplatin-induced kidney injury^24,25^. The fact that FSP1 induces the expression of these four factors in mProx cells indicates that Nrf2 activation is essential for the renoprotection observed in this study. RAGE is a well-known FSP1 receptor. Our findings indicate that the protective effects of FSP1 on TEC and its renoprotection observed in mice with cisplatin nephrotoxicity were RAGE-dependent. However, FSP1 weakly induced these four factors even in RAGE^−/−^ mProx cells, suggesting that an unknown independent pathway of RAGE is also likely present.

In this study, we observed that FSP1 ameliorates acute tubular injury in mice with cisplatin nephrotoxicity. Cisplatin is one of the most commonly used and most effective anti-tumor agents. However, the development of cisplatin nephrotoxicity is an inevitable complication and an obstacle to its extended use in chemotherapy. Although our findings suggest that FSP1 may be an effective agent for treating cisplatin induced acute kidney injury, several reports focusing on the function of extracellular FSP1 indicate that it acts as a pathological aggravating factor in cancer with the potential to induce metastasis. Consequently, its clinical application can be difficult, given the need for relatively high doses (20 μM) and the possibility of unwanted side effects.

We also demonstrated that tubular injury is improved in FSP1.TG mice, where FSP1 is specifically overexpressed in podocytes. This beneficial effect is canceled in mice crossed with *Ager*^−/−^ mice. Similar renoprotective effects against cisplatin nephrotoxicity were observed in mice intraperitoneally injected with recombinant FSP1, suggesting that extracellular FSP1 secreted from podocytes binds to RAGE in TECs, thus protecting these cells. Therefore, it is reasonable to hypothesize that podocyte FSP1 observed in human active glomerulonephritis may protect TEC within the same nephron.

However, the mechanism underlying FSP1 secretion is still unknown^2^. Because it lacks a signal peptide, FSP1 cannot be secreted through the usual endoplasmic reticulum-Golgi-dependent pathway. On the other hand, it has been reported that some soluble proteins are transported into the endoplasmic reticulum despite the absence of a signal peptide^26,27^ and that various cytokines mediate FSP1 secretion in different types of normal and tumor cells^28,29^. In the present study, increased expression of two Nrf2-related factors (CSE and sqstm1) was not observed in FSP1.TG mice, which suggests FSP1 secretion may be too low to induce overproduction of CSE and sqstm1 under normal conditions. Our hypothesis is that unknown factors induced by cisplatin nephrotoxicity may mediate FSP1 secretion in podocytes, but this is not beyond speculation. In addition, we cannot completely rule out the possibility that overexpression of FSP1 in podocytes induces some functional changes in podocytes, resulting indirectly in improvement of tubular injury. This is a limitation of our study.

In conclusion, extracellular FSP1, which can be excreted by podocytes, has Nrf2-mediated antioxidant activity and exerts renoprotective effects by protecting the tubular epithelium from ROS. From this mouse model, the existence of direct interaction between podocytes and tubular epithelium was suggested.

## Methods

### Reagents

Human recombinant FSP1, which is 93% identical to mouse FSP1, was generated as previously described^7^. Rabbit polyclonal anti-human FSP1 antibody (1:5000 dilutions), mouse anti-human FSP1 monoclonal antibody (1:2000 dilutions), and rabbit polyclonal anti-mouse FSP1 (1:5000 dilutions) were generated as previously described^5–7^. The goat polyclonal synaptopodin antibody (P-19) (sc-21537) (1:5000 dilutions) was purchased from Santa Cruz Biotechnology (US). Antibodies against AKT (4691S) (1:1000 dilutions), pAKT (4060S) (1:1000 dilutions), Lamin A/C (2032S) (1:1000 dilutions), and cleaved caspase-3 (9661 for western blot, 9664 for immunohistochemistry) (1:1000 dilutions) were purchased from Cell Signaling Technology (US). An antibody against Nrf2 (BS1074R) (1:1000 dilutions) was purchased from Bioss (US), and an antibody against β-actin (ab8227) (1:1000 dilutions) was purchased from Abcam, (UK). Nonspecific reactions were blocked with blocking reagent (X0909), purchased from Agilent (US). Cisplatin was purchased from SIGMA-Aldrich (US).

### Cell culture

Mouse proximal tubular epithelial (mProx) cells were provided by Dr. Takeshi Sugaya of St. Marianna University School of Medicine. mProx cells were cultured in Dulbecco's Modified Eagle Medium (DMEM) with 10% FCS at 37 °C in a humid environment with 5% CO_2_/95% air^30^. To achieve semi-confluence, mProx cells (passages 10 through 14) were seeded in 24-well plates and the medium was changed every two days thereafter. To investigate the effects of FSP1, mProx cells were treated for 2 h with FSP1 at a concentration of 20 μM for Western blotting, or for 12 h with FSP1 at a concentration of 20 μM for real-time PCR.

### Mice

All animal experiments, which were conducted in compliance with the National Research Council’s Guide for the Care and Use of Laboratory Animals, were approved by the Fukui University Animal Care Committees (Approval number; R05029), and followed the recommendations in the ARRIVE guidelines. All mice were raised in standard cages under a controlled 12-h light/dark cycle at 22 ± 2 °C and were allowed free access to water and chow. Male C57BL/6 J mice (8–12 weeks of age) were injected intraperitoneally with 20 mg/kg cisplatin to generate the cisplatin nephrotoxicity model. Thirty minutes before cisplatin injection, mice received isotonic saline only (control) or intraperitoneal doses of FSP1 (50 μg/g) dissolved in isotonic saline. The mice were then euthanized 72 h later^31^. To generate the transgenic mice in which podocytes expressed FSP1, a transgene consisting of the promoter of the mouse nephrin gene, mouse FSP1, and the polyA signal from human growth hormone was injected into fertilized eggs obtained after BDF1x C57BL/6N mating. The transgenic mouse line was maintained by backcrossing with the C57BL/6J strain for more than 12 generations. The receptors for advanced glycation end products (RAGE) knockout mice (*Ager*^−/−^) were generated as previously described^32^ and backcrossed with the C57BL/6J strain for more than 12 generations. Male mice were also used to generate the cisplatin nephrotoxicity in genetically modified mice. All mice euthanasia was performed by overdose of isoflurane.

### Microarray analysis

mProx cells were left untreated or treated with FSP1 (20 μM) for 12 h before total RNA was extracted using a MagExtractor RNA kit (TOYOBO CO. LTD, Japan). An Agilent Bioanalyzer was used to check the integrity of the RNA. Using a GeneChip WT Plus Reagent Kit, sense-strand DNA was produced from 200 ng of total RNA and then fragmented and labeled (Thermo Fisher Scientific, Japan). Gene expression profiles in mProx cells after FSP1 treatment were clarified using the Clariom S assay microarray. The scatter plot was created to visualize the differences in the expressed genes using Transcriptome Analysis Console software (Thermo Fisher Scientific, Japan).

### Quantitative real-time polymerase chain reaction (qPCR)

As previously reported^33^, TaqMan real-time PCR experiments were performed. Targets for mouse heme oxygenase 1 (Mm00516005_m1), mouse solute carrier family 7 member 11 (Mm00442530_m1), mouse cystathionine gamma-lyase (Mm00461247_m1), mouse sequestosome 1 (Mm00448091_m1), and mouse RN18-S (Mm03928990_g1) were purchased from Thermo Fisher Scientific as unlabeled specific primers and TaqMan MGB probes (6-FAM dye-labeled). The levels of mRNA for each gene were scaled to those of 18S rRNA. A value of 1.0 was assigned to the average quantity of mRNA for each gene in the untreated cells.

### Gene knockout using the CRISPR/Cas9 system

crRNA was designed to target exon 2 of the Mus musculus *Ager* gene (target sequence, 5ʹ-GATTGGAGAGCCACTTGTGC-3ʹ). To prepare Cas9 gRNAs, equimolar amounts of Alt-R™ crRNA and Alt-R tracrRNA (Integrated DNA Technologies, US) were mixed in IDT Duplex Buffer (30 mM HEPES, pH 7.5, 100 mM potassium acetate; Integrated DNA Technologies), heated to 95 °C, and then slowly cooled to room temperature. To generate the RNP complex, a mixture of gRNAs, diluted Cas9 enzyme, and Opti-MEM^®^ (Thermo Fisher Scientific) was incubated for 5 min at room temperature. Lipofection was then performed in 96-well plates. Opti-MEM^®^ containing Lipofectamine^®^ RNAiMAX (Thermo Fisher Scientific) was combined with an equal volume of Opti-MEM containing RNP and incubated for 20 min at room temperature. After lipoplex formation, 3 × 10^5^ mProx cells resuspended in 1 mL of DMEM + 10% FBS were added to the transfection complex. Transfection plates were incubated at 37 °C under 5% CO_2_/95% air. Mutation was confirmed using a Guide-it™ Genotype Confirmation Kit (TAKARA, Japan) and Sanger sequences. Fourteen bases of the target gene were deleted and the terminal codon was inserted (5ʹ-GCCACTTGTGCTAAGCTGTAA-3ʹ).

### Western blotting

mProx were lysed in RIPA buffer containing phosphatase inhibitors (SIGMA-Aldrich). The nuclei extraction of mProx cells was performed with a Nuclear Extract Kit (Active Motif, Japan) according to the manufacturer’s protocol. Protein levels in cell and nuclear lysates were quantified using the Lowry method with a Bio-Rad DC™ protein assay kit (Bio-Rad Laboratories, U. S.), after which samples were prepared in cracking buffer such that each sample had the same amount of protein. Protein separation was performed on a polyacrylamide gel (gradient gel containing 10–20% acrylamide), and the blots were transferred to nitrocellulose membranes (Immun-Blot PVDF Membrane; Bio-Rad Laboratories), after which the membranes were hybridized with a peroxidase-labeled secondary antibody. SuperSignal™ West Dura Extended Duration Substrate was used to create a fluorescence signal, which was then viewed with an Image Quant LAS 4000 (GE Healthcare Life Sciences, US). Signal intensities were corrected with endogenous control proteins using Image J software (NIH, US).

### Determination of intracellular ROS levels

According to the manufacturer's instructions, the OxiSelect™ Intracellular ROS Assay Kit (Cell Biolabs, Inc., US) was used to quantify the levels of intracellular ROS.

### Measurement of reduced glutathione (GSH) and oxidized glutathione (GSSG)

GSH (reduced glutathione) and GSSG (oxidized glutathione) levels were measured using a GSSG/GSH Quantification Kit (Dojindo Molecular Technologies, Inc, Japan).

### Flow cytometry

An Annexin V-FITC kit (MBL CO. LTD., Japan) was used in accordance with the manufacturer's instructions to detect translocation of phosphatidylserine from the inner to the outer plasma membrane (a sign of early apoptosis). Briefly, mProx cells grown in 12 well plates were treated for 24 h with or without cisplatin (25 μM) in the presence or absence of FSP1 (20 μM). The cells were then trypsinized, rinsed with phosphate-buffered saline, and incubated in binding buffer containing annexin V and PI for 15 min at room temperature in the dark. Fluorescence-activated cell sorting (BD FACSCanto II, BD Biosciences, United States) and CellQuest software (BD Biosciences) were used for flow cytometry analysis of the labeled cells. 10,000 events/sample were collected in total to ensure that there was enough data.

### Histopathology, tubular injury score, TUNEL, cleaved caspase-3 staining, and FSP1 staining

Formalin-fixed, paraffin-embedded mouse kidney tissues were cut into sections and stained with periodic acid-Schiff (PAS) for histopathological analysis. Tubular injury was evaluated in PAS-stained kidney sections and assigned a score of 0–5 based on the percentage of damaged tubules, which were identified by the presence of diffuse tubular dilatation, intraluminal casts, and/or tubular cell blebbing, vacuolization and detachment: 0, normal; 1, 1–10%; 2, 11–25%; 3, 26–45%, 4, 46–75%; and 5, 76–100%. The slides were scored in a blinded manner and the results are means of 10 representative fields/group^34^. Dual immunofluorescent staining was carried out as previously described^9^ using rabbit polyclonal anti-mouse FSP1 antibody (1:5000 dilution) and goat polyclonal anti-mouse synaptopodin antibody (1:500 dilution; Santa Cruz Biotechnology Inc.).

Using a commercial kit (TAKARA), TUNEL was performed after each of the aforementioned treatments. The number of TUNEL-positive cells and the total number of cells (nuclei) in four to six photomicrographs (200 magnification, around 1500 cells) were counted by a blinded observer to calculate the proportion of apoptotic cells. This experiment was performed three times on different days. An average of three independent experiments were used to express the apoptosis rate.

Formalin-fixed, paraffin-embedded sections were dewaxed, rehydrated, and microwave heated for 20 min in antigen retrieval solution. Endogenous peroxidase was blocked for 20 min (S2023, Agilent, US) after three consecutive 5-min washes in TBS. Nonspecific reactions were blocked with blocking reagent (X0909, Agilent) for 15 min. The sections were incubated with each primary antibody (rabbit polyclonal anti-mouse FSP1, anti-cleaved caspase-3) at 4 °C overnight, then washed for 5 min with TBS and incubated for 30 min with Histofine simple stain mouse MAX-PO (R) (414341, Nichrei, Japan). The sections were then washed again for 5 min with TBS. Diaminobenzidine (425011, Nichrei) was used as the chromogen. Counterstaining was with hematoxylin.

HistoQuest Ver. 7.1 (TissueGnostics GmbH, Austria) was used for the quantitative analysis of FSP1 and cleaved caspase-3 immunostaining. Five fields of view (0.3 × 0.4 µm) of intensely stained regions in the renal cortex were selected for evaluation. The analytical options used were total area measurements for the segmentation method and single reference shade V2 for the color separation method. In automatic detection mode, brown tones were automatically color resolved to 0–255 color shades, and positive areas (µm^2^) were determined with a threshold of 40–255 (FSP1) or 5–255 (cleaved caspase-3). Positive areas in the 5 fields of view were then summed.

### Determination of serum creatinine (Scr), serum blood urea nitrogen (BUN), and urinary albumin concentrations

Concentrations of Scr and BUN in mouse blood and urine samples were determined in duplicate using standard enzymatic methods (Cygnus Auto CRE, Shino-Test Corp., Tokyo, Japan and Aqua-auto Kainos UN-II Test Kit, Kainos, Tokyo, Japan, respectively) with a Toshiba 2000FR analyzer (Toshiba Medical Systems Corp., Tokyo, Japan) in a central laboratory. Urine was collected for 24 h in metabolic cages, after which commercial kits were used to measure urinary concentrations of albumin (Albuwell M, Exocell, US).

### Statistical analysis

Statistical calculations were performed using SigmaPlot 15 (Systat Software, Inc., San Jose, CA). We used the Mann–Whitney rank test for skewed distributions and the paired t-test (P values were two-tailed for assay) for data with a normally distributed distribution to compare two groups. We utilized the Holm-Sidak method and one-way analysis of variance (ANOVA) for multiple comparisons of normally distributed data when comparing three or more groups, and the Kruskal–Wallis one-way ANOVA on ranks for comparing multiple comparisons of skewed distributions. Values of P < 0.05 were considered significant.

### Supplementary Information

Supplementary Information.

## Data Availability

Data will be made available from the corresponding author on reasonable request.
