# Supplementary material for: Renoprotective effects of extracellular fibroblast specific protein 1 via nuclear factor erythroid 2-related factor-mediated antioxidant activity
Source: Sci Rep. 2023 Dec 18;13:22540. doi: 10.1038/s41598-023-49863-y (PMC10728167; doi:10.1038/s41598-023-49863-y)
Supplement: Supplementary file 1 — Supplementary Information. [file 41598_2023_49863_MOESM1_ESM.pptx]

## Slide 1
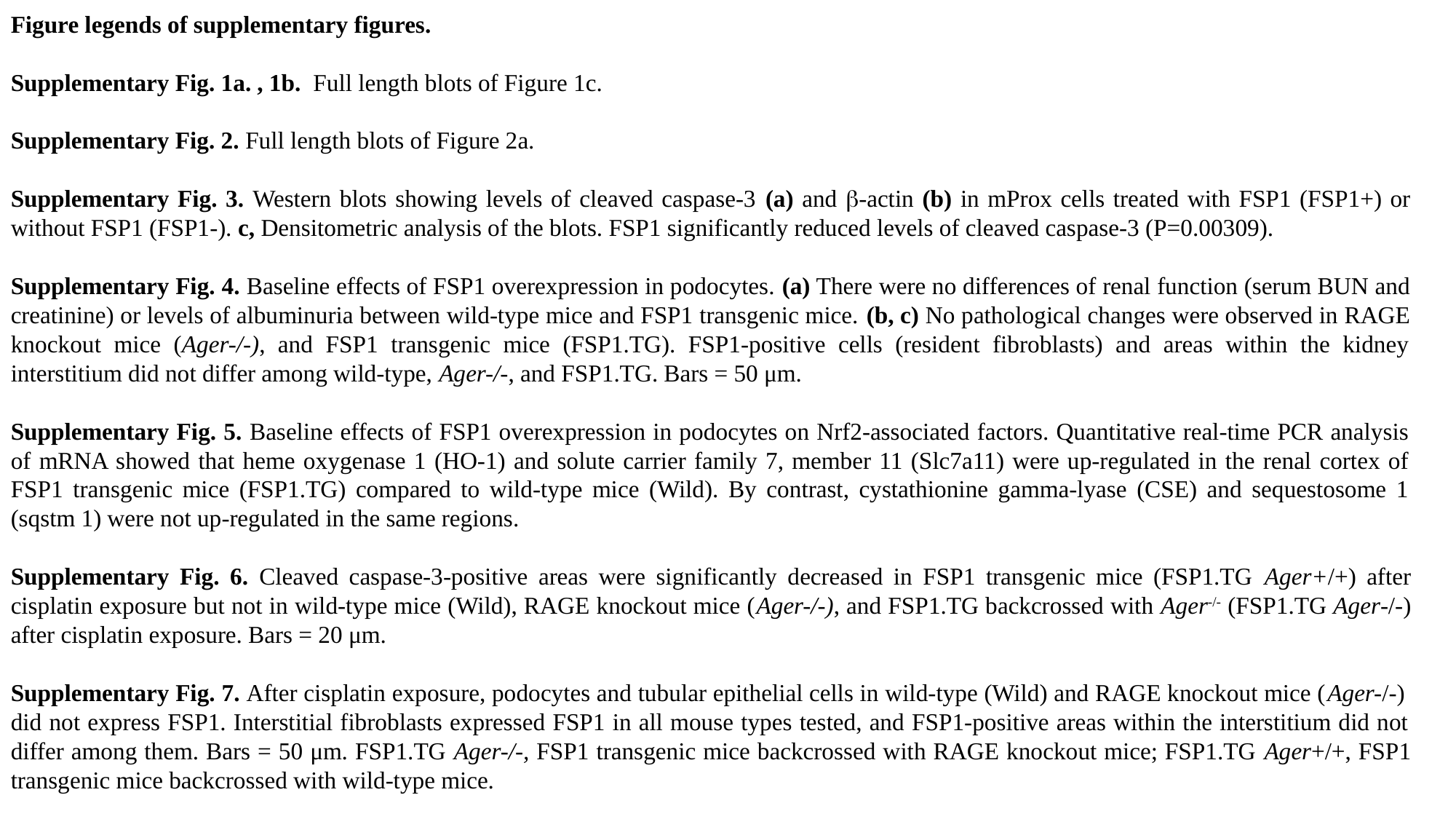

Figure legends of supplementary figures.
Supplementary Fig. 1a. , 1b. Full length blots of Figure 1c.
Supplementary Fig. 2. Full length blots of Figure 2a.
Supplementary Fig. 3. Western blots showing levels of cleaved caspase-3 (a) and β‑actin (b) in mProx cells treated with FSP1 (FSP1+) or without FSP1 (FSP1-). c, Densitometric analysis of the blots. FSP1 significantly reduced levels of cleaved caspase-3 (P=0.00309).
Supplementary Fig. 4. Baseline effects of FSP1 overexpression in podocytes. (a) There were no differences of renal function (serum BUN and creatinine) or levels of albuminuria between wild-type mice and FSP1 transgenic mice. (b, c) No pathological changes were observed in RAGE knockout mice (Ager-/-), and FSP1 transgenic mice (FSP1.TG). FSP1-positive cells (resident fibroblasts) and areas within the kidney interstitium did not differ among wild-type, Ager-/-, and FSP1.TG. Bars = 50 μm.
Supplementary Fig. 5. Baseline effects of FSP1 overexpression in podocytes on Nrf2-associated factors. Quantitative real-time PCR analysis of mRNA showed that heme oxygenase 1 (HO-1) and solute carrier family 7, member 11 (Slc7a11) were up-regulated in the renal cortex of FSP1 transgenic mice (FSP1.TG) compared to wild-type mice (Wild). By contrast, cystathionine gamma-lyase (CSE) and sequestosome 1 (sqstm 1) were not up-regulated in the same regions.
Supplementary Fig. 6. Cleaved caspase-3-positive areas were significantly decreased in FSP1 transgenic mice (FSP1.TG Ager+/+) after cisplatin exposure but not in wild-type mice (Wild), RAGE knockout mice (Ager-/-), and FSP1.TG backcrossed with Ager-/- (FSP1.TG Ager-/-) after cisplatin exposure. Bars = 20 μm.
Supplementary Fig. 7. After cisplatin exposure, podocytes and tubular epithelial cells in wild-type (Wild) and RAGE knockout mice (Ager-/-) did not express FSP1. Interstitial fibroblasts expressed FSP1 in all mouse types tested, and FSP1-positive areas within the interstitium did not differ among them. Bars = 50 μm. FSP1.TG Ager-/-, FSP1 transgenic mice backcrossed with RAGE knockout mice; FSP1.TG Ager+/+, FSP1 transgenic mice backcrossed with wild-type mice.

## Slide 2
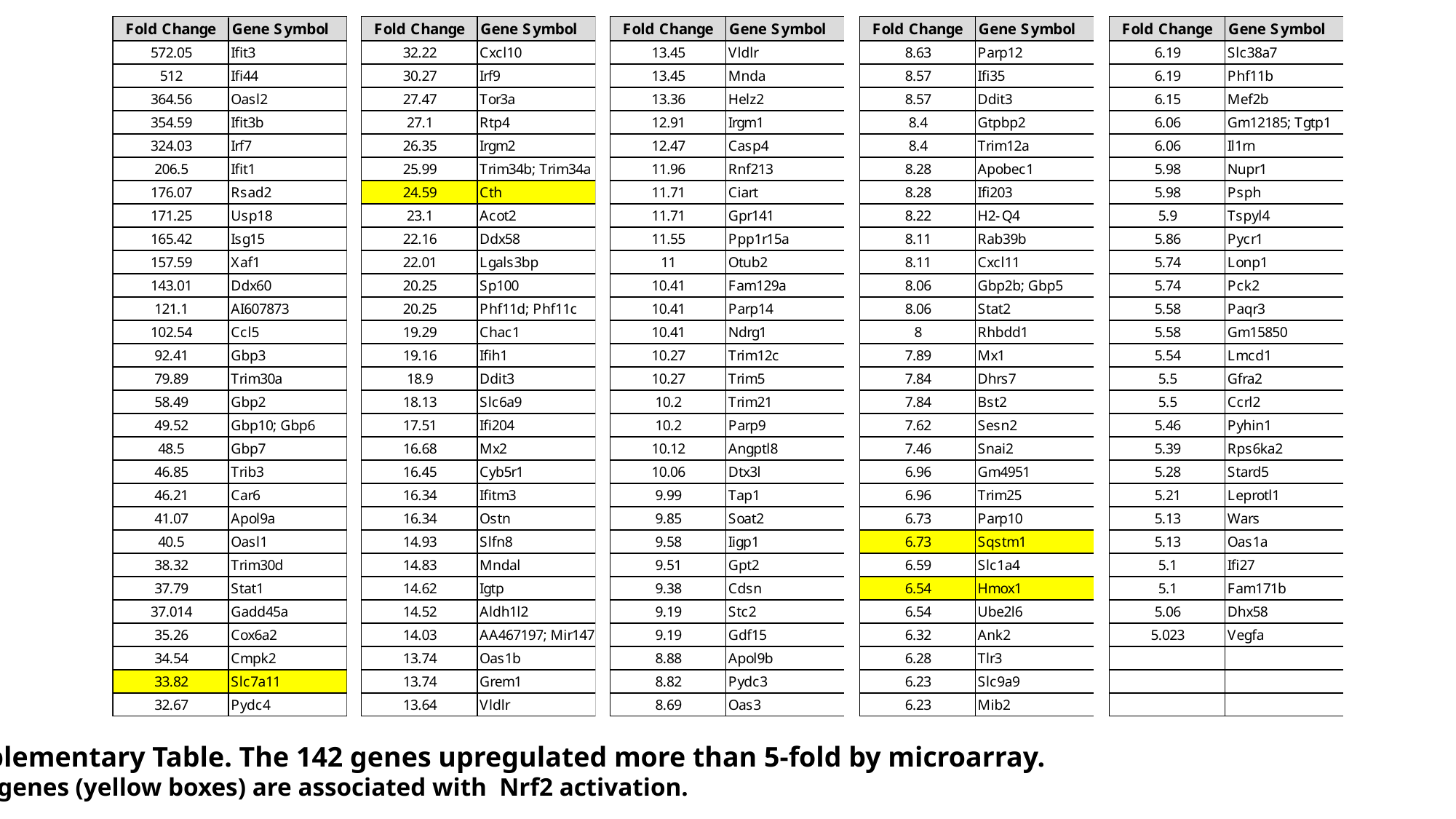

Supplementary Table. The 142 genes upregulated more than 5-fold by microarray.
Four genes (yellow boxes) are associated with Nrf2 activation.

## Slide 3
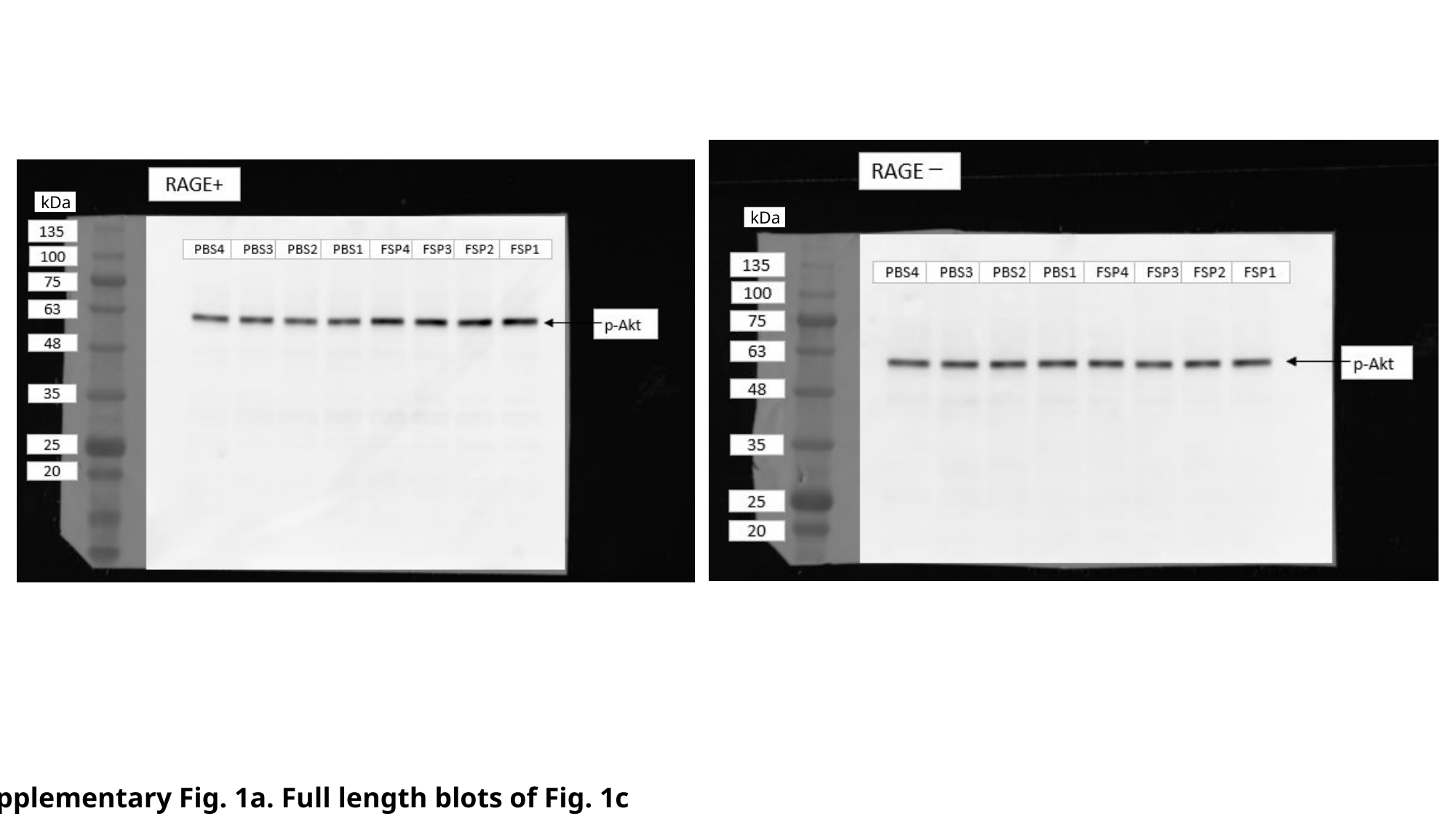

kDa
kDa
Supplementary Fig. 1a. Full length blots of Fig. 1c

## Slide 4
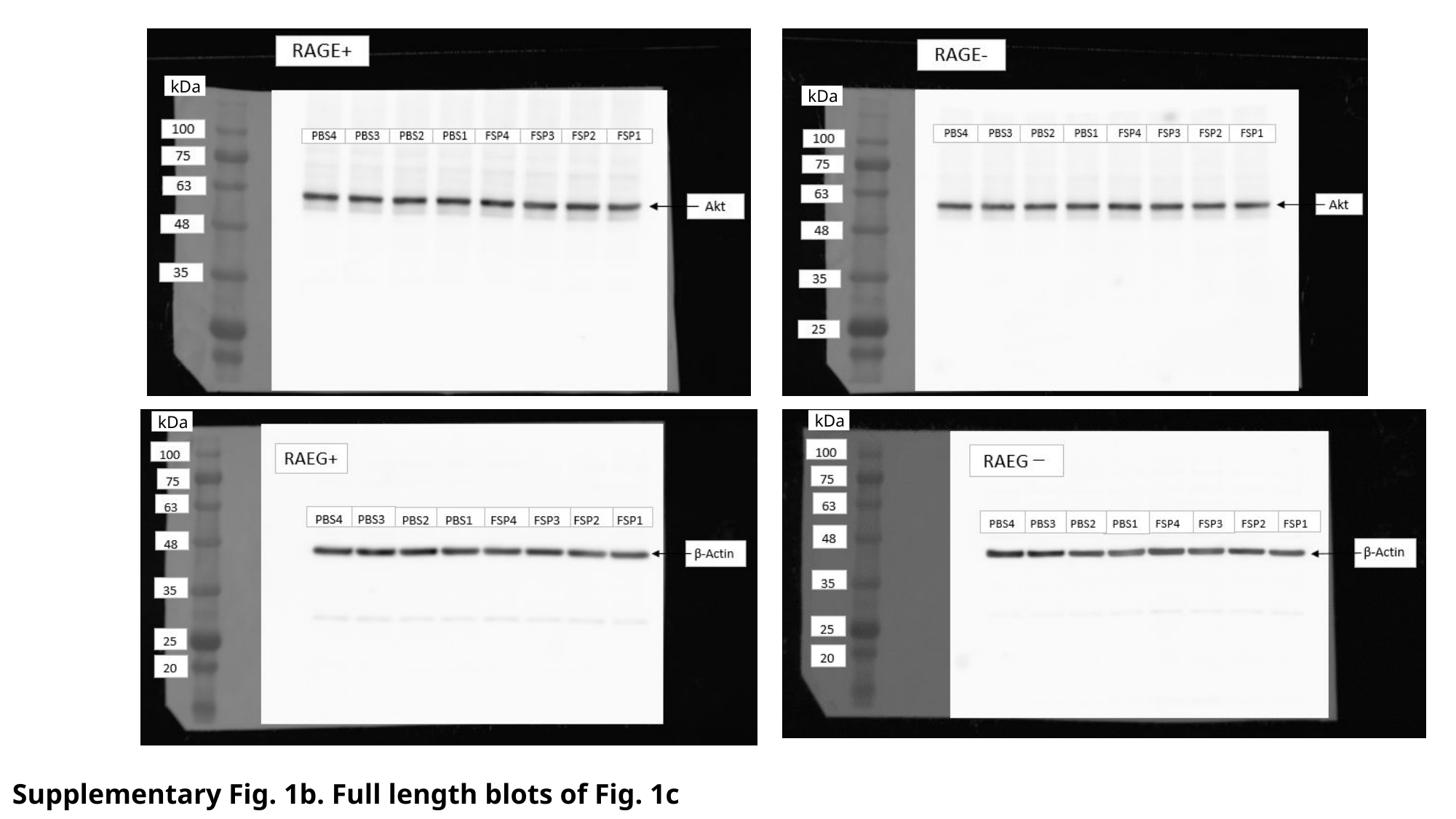

kDa
kDa
kDa
kDa
Supplementary Fig. 1b. Full length blots of Fig. 1c

## Slide 5
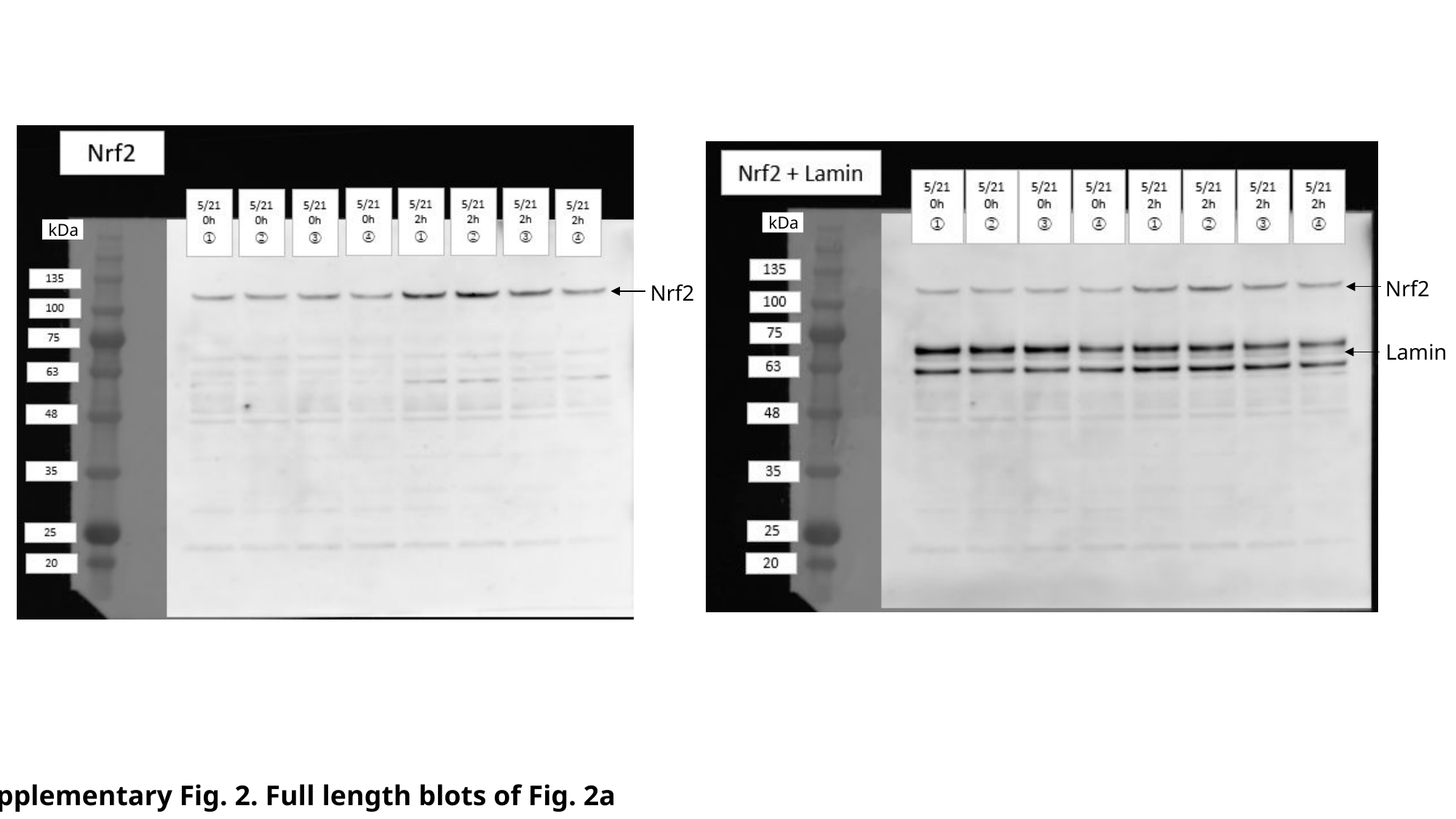

kDa
kDa
Nrf2
Nrf2
Lamin
Supplementary Fig. 2. Full length blots of Fig. 2a

## Slide 6
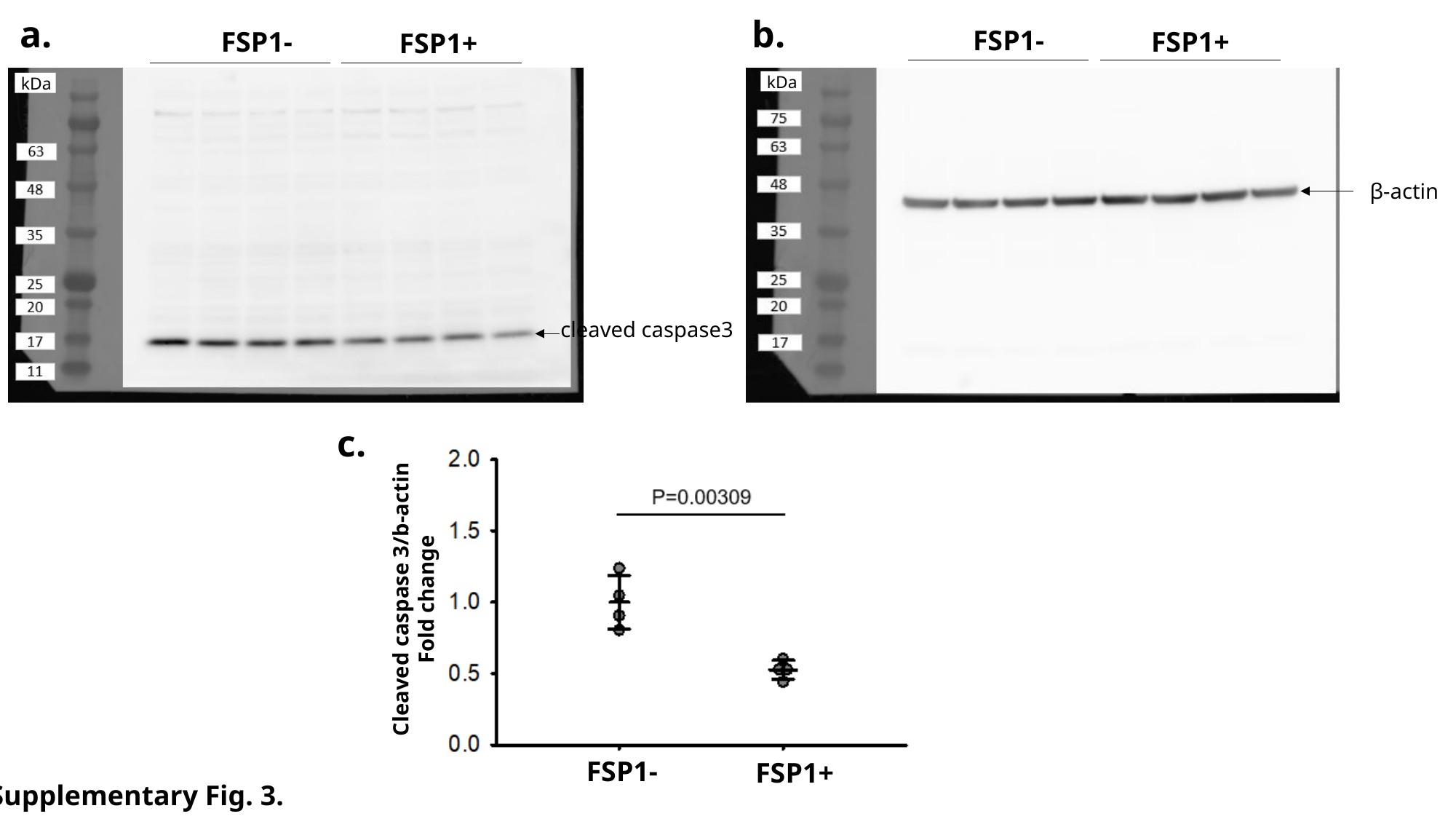

a.
b.
FSP1-
FSP1-
FSP1+
FSP1+
kDa
kDa
β-actin
cleaved caspase3
c.
Cleaved caspase 3/b-actin
Fold change
FSP1-
FSP1+
Supplementary Fig. 3.

## Slide 7
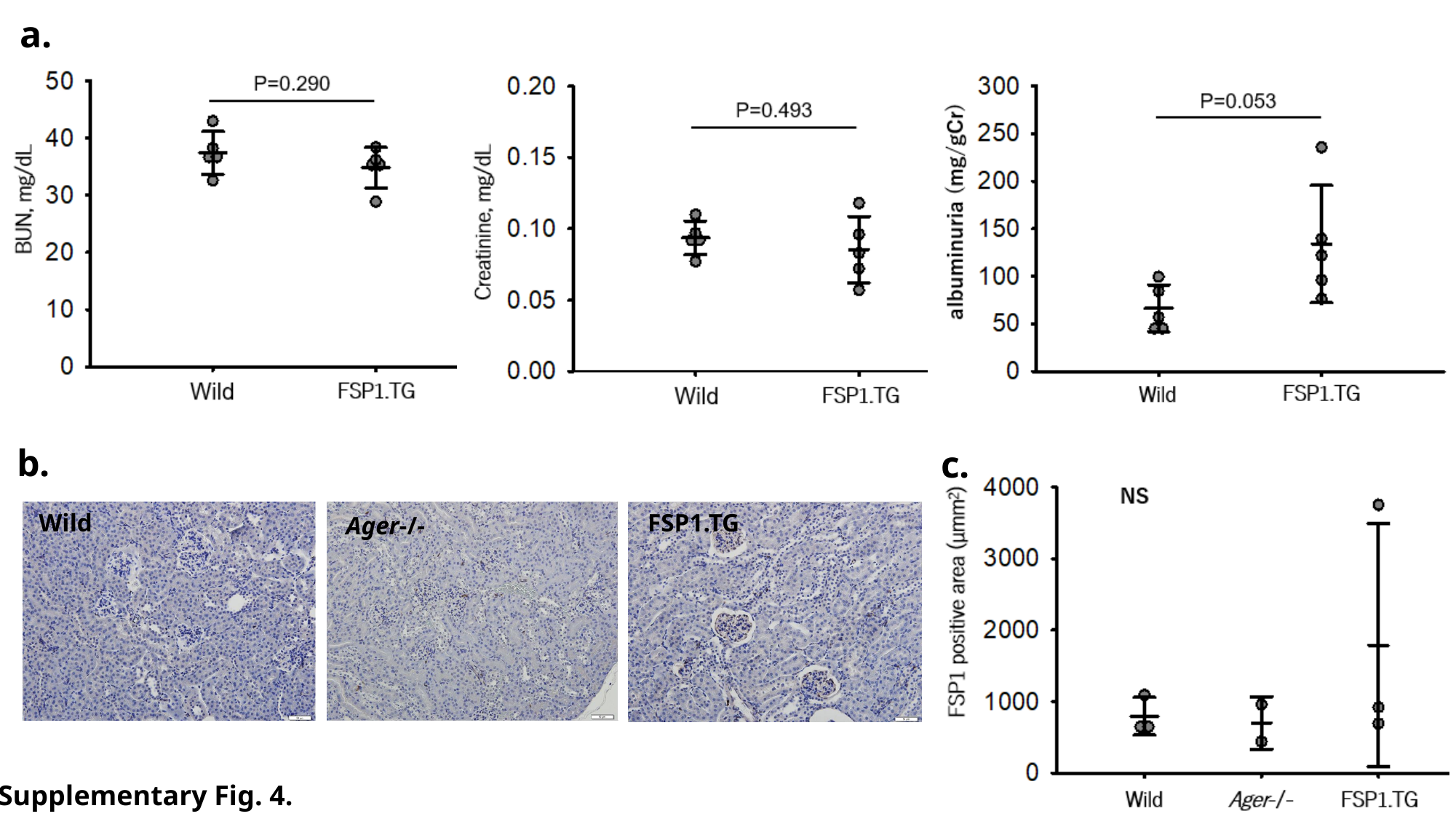

a.
b.
c.
Wild
FSP1.TG
Ager-/-
Supplementary Fig. 4.

## Slide 8
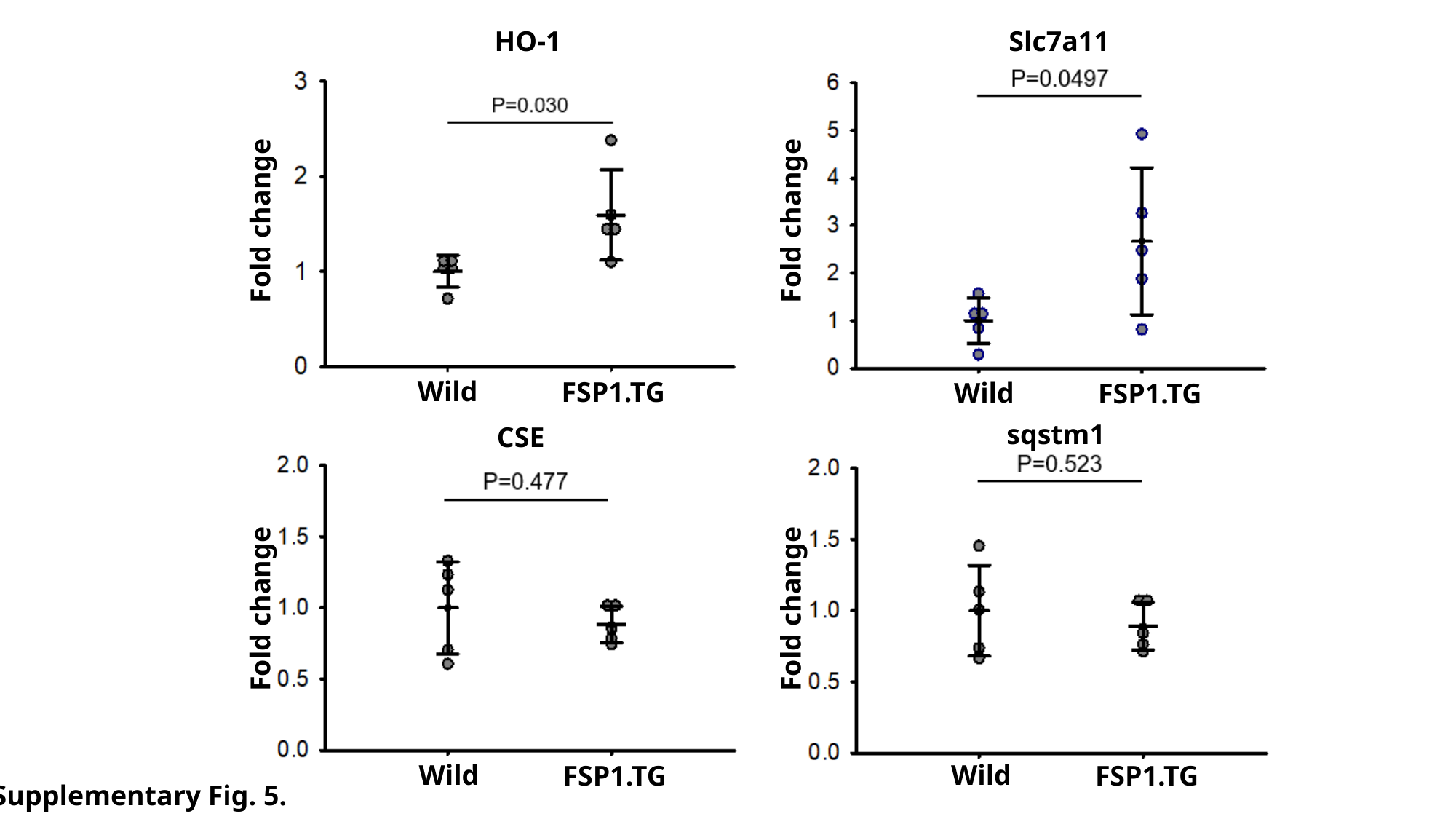

HO-1
Slc7a11
Fold change
Fold change
Wild
FSP1.TG
Wild
FSP1.TG
sqstm1
CSE
Fold change
Fold change
Wild
Wild
FSP1.TG
FSP1.TG
Supplementary Fig. 5.

## Slide 9
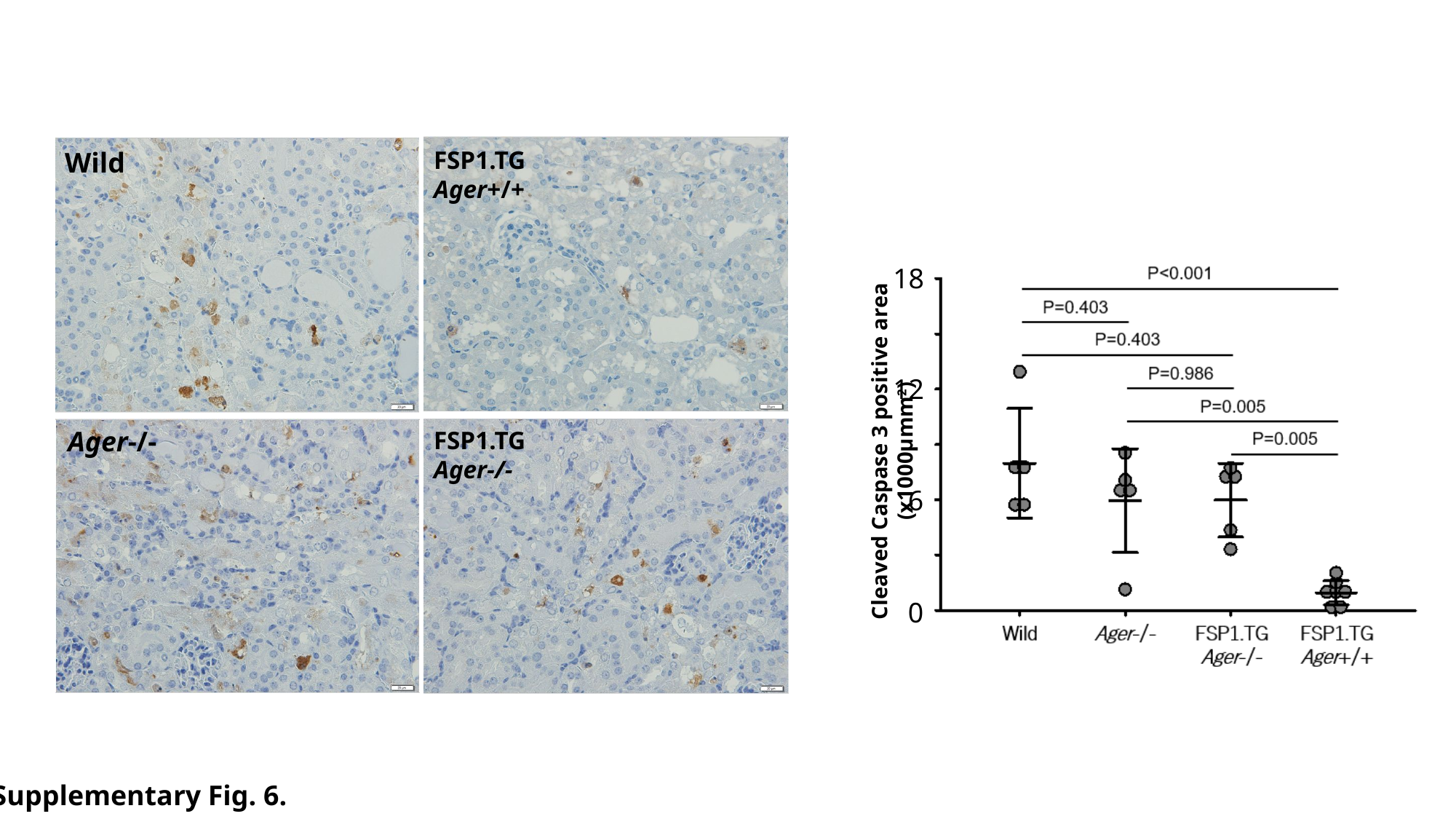

FSP1.TG
Ager+/+
Wild
18
12
Ager-/-
FSP1.TG
Ager-/-
Cleaved Caspase 3 positive area (x1000µmm2)
6
0
Supplementary Fig. 6.

## Slide 10
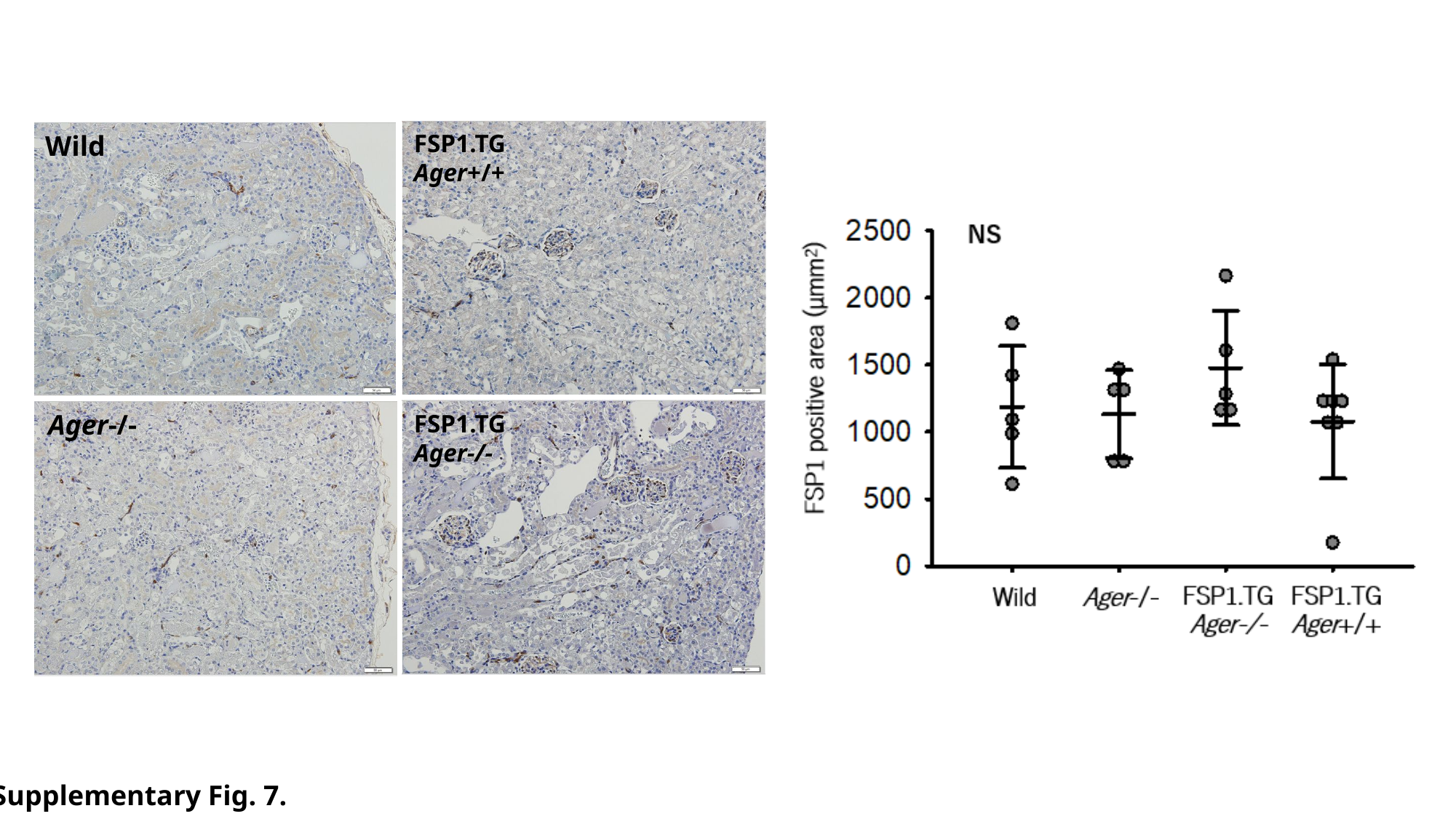

FSP1.TG
Ager+/+
Wild
Ager-/-
FSP1.TG
Ager-/-
Ager-/-
Supplementary Fig. 7.
